# Molecular and clinical analysis of *TRPC6* and *AGTR1* genes in patients with pulmonary arterial hypertension

**DOI:** 10.1186/s13023-014-0216-3

**Published:** 2015-01-21

**Authors:** Guillermo Pousada, Adolfo Baloira, Diana Valverde

**Affiliations:** Department Biochemistry, Genetics and Immunology, Faculty of Biology, University of Vigo, Campus As Lagoas Marcosende S/N, 36310 Vigo, Spain; Instituto de Investigación Biomédica de Vigo (IBIV), Vigo, Spain; Complexo Hospitalario Universitario de Pontevedra, Servicio de Neumología, Pontevedra, Spain

**Keywords:** Pulmonary Arterial Hypertension, *TRPC6*, *AGTR1*, Polymorphism, Correlation genotype/phenotype

## Abstract

**Background:**

Pulmonary arterial hypertension (PAH) is a rare and progressive vascular disorder characterized by increased pulmonary vascular resistance and right heart failure. The aim of this study was to analyze 5′UTR region in canonical transient receptor potential isoform 6 (*TRPC6*) and 3′UTR region in Angiotensin II type I receptor (*AGTR1*) genes in patients with idiopathic and associated PAH. Correlation among mutations and clinical and functional parameters was further analyzed.

**Methods:**

Analysis of *TRPC6* and *AGTR1* genes was performed by polymerase chain reaction (PCR) and direct sequencing. We used a non-parametric test to determine if significant differences were found between the groups studied and chi-square test to compare clinical and hemodynamic variables among genotypes.

**Results:**

Fifty five patients and fifty two controls were included in this study. We found statistically significant differences for c.1-361A > T (p = 0.0077), c.1-254C > G (p < 0.0001) and c.1-218C > T (p = 0.0021) in *TRPC6* gene and c.1166A > C (p < 0.001) in *AGTR1* gene, between patients and controls. Idiopathic PAH patients (IPAH) and controls presented significant differences for all 3 *TRPC6* polymorphisms (p = 0.020), (p = 0.002) and (p = 0.008) respectively, and also showed differences for *AGTR1* gene (p < 0.001). In associated PAH (APAH) patients we found statistical differences for c.1-254C > G (p < 0.001) and c.1-218C > T (p = 0.001) in *TRPC6* gene and c.1166A > C (p = 0.001) in *AGTR1* gene. Several clinical and hemodynamic parameters showed significant differences between carriers and non-carriers of these single nucleotide polymorphisms (SNPs). Nineteen patients were carriers of all 3 SNPs in *TRPC6* gene and presented a more severe phenotype with differences in mean pulmonary arterial pressure (p = 0.016), systolic pulmonary arterial pressure (p = 0.040), cardiac index (p < 0.001) and 6 minute walking test (p = 0.049). 16 of these patients harbored the SNP in *AGTR1* gene. These patients showed differences in age at diagnosis (p = 0.049), mean pulmonary arterial pressure (p = 0.033), cardiac index (p = 0.002) and 6 minute walking test (p = 0.039).

**Conclusions:**

PAH is a rare disease with pulmonary vascular remodeling caused in part by a heterogeneous constellation of genetic arrangements. This study seems to suggest that c.1-361A > T, c.1-254C > G and c.1-218C > T polymorphisms in *TRPC6* gene and c.1166A > C polymorphism in *AGTR1* could have a role in the development of this disease.

## Background

Pulmonary arterial hypertension (PAH; OMIM 178600, ORPHA 422) is a rare and progressive vascular disorder characterized by increased pulmonary vascular resistance and right heart failure. It is diagnosed when mean pulmonary arterial pressure (mPaP) at rest is ≥ 25 mmHg with a pulmonary arterial wedge pressure (PAWP) ≤ 15 mmHg [1]. Symptoms of PAH include fatigue, shortness of breath and syncope. The estimated incidence is approximately 2–5 cases per million per year. For adults, mean age at presentation ranges from 36 to 50 years, although individuals at any age could be affected [2]. The disease is more frequent in women, with a ratio of at least 1.7:1 [3]. Progression of pulmonary hypertension leads to right ventricular failure and death in three years from diagnosis without treatment [4]. PAH can be inherited (HPAH), idiopathic (IPAH), or associated with other diseases, drug or toxin exposures (APAH) [5]. When familial aggregation or a genetic defect has been identified in IPAH patients, they will be classified as HPAH [6]. Much of what is known about the genetic basis of PAH is related to mutations in bone morphogenetic protein receptor 2 (*BMPR2*) [7-9]. Patients harboring *BMPR2* mutations have some differences in clinical course and outcomes [10]. However, phenotypic expression of PAH is highly variable, probably related to environmental factors, other genes and/or genetic modifiers [11-13].

In human pulmonary artery smooth muscle cells (PASMCs), the canonical transient receptor potential (*TRPC*) channel genes are involved in agonist-mediated pulmonary vasoconstriction and mitogen-mediated cell proliferation [14]. Of the seven *TRPC* (*TRPC1–7*) isoforms identified, recently it has been demonstrated that only *TRPC1*, *TRPC4*, and *TRPC6* are expressed in distal pulmonary artery smooth muscle and PASMCs [15,16]. *TRPC6* is an important isoform highly expressed in the lungs and pulmonary arteries. *TRPC6* upregulation is also a critical initial step in the increase of [Ca_2_^+^]_cyt_ required for mitogen-mediated PASMC proliferation and a key contributor to the elevated [Ca_2_^+^]_cyt_ in PAH PASMC [17,18]. The c.1-361A > T (rs41302375), c.1-254C > G (rs3824934) and c.1-218C > T (rs56134796) polymorphisms located in 5′-untranslated region (UTR) may be involved in the development of PAH [19].

Angiotensin II is a potent vasopressor hormone and a primary regulator of aldosterone secretion. It is an important factor controlling blood pressure and an essential effector on the regulation of cardiovascular homeostasis. Angiotensin II type I receptor (*AGTR1*) plays an important role in blood pressure control and is implicated in the pathogenesis of coronary diseases [20,21]. Distal muscularization in pulmonary vessels induced by chronic hypoxia is associated with an early and transient increase in *AGTR1* indicating that the vasotonic response to angiotensin II is mainly due to this receptor [22]. The c.1166A > C (rs5186) polymorphism in the 3′-UTR of the gene has been extensively studied in hypertension [23,24] and has been related to later age at diagnosis in IPAH suggesting a role in the phenotypic variability of this disease [25].

The aim of this study was to analyze three SNPs located in 5′UTR region in *TRPC6* gene and one SNP in 3′UTR region in *AGTR1* gene, in consecutive patients with IPAH and APAH, and to search for associations with clinical and hemodynamic variables.

## Methods

### Patients and samples

Patients with idiopathic or associated PAH (group 1 of the new classification of Nice) [5] followed in our PAH Unit were included in this study. Cardiac catheterization was performed using the latest consensus diagnostic criteria of the ERS-ESC (mean resting pulmonary pressure ≥25 mmHg, PAWP <15 mmHg) in all cases [26]. PAH was considered idiopathic after exclusion of any of the possible clinical causes associated with the disease. Clinical history included use of drugs, especially appetite suppressants, and screening for connective tissue and hepatic diseases. The study included serology for HIV, autoimmunity, thoracic CT scan and echocardiography. Patients with chronic lung disease that could be related to PAH, were excluded. Fifty two healthy individuals were used as controls. All patients and controls signed an informed consent. The Autonomic Ethics Committee approved the study (*Comité Autonómico de Ética da Investigación de Galicia - CAEI de Galicia*).

### Identification of SNPs in TRPC6 and AGTR1 genes

Genomic DNA was extracted from leukocytes isolated from venous blood using the FlexiGene DNA Kit (Qiagen, Germany) according to the manufacturer’s protocol. We performed a PCR with 0.2 U of Taq Polymerase (Biotaq, Bioline, UK) and 0.75 mM Cl_2_Mg for the c.1-361A > T, c.1-254C > G and c.1-218C > T polymorphisms in *TRPC6* gene and c.1166A > C polymorphism in *AGTR1* gene. Amplification conditions were as follows: 95°C for 5 min, 35 cycles of 95°C for 1 min, 60°C for 1 min, 72°C for 1 min and finally, 72°C for 10 min for *TRPC6* gene and 95°C for 5 min, 35 cycles of 95°C for 30 s, 58°C for 30 s, 72°C for 30 s and finally, 72°C for 7 min for *AGTR1* gene. The forward and reverse primers were 5′-AGAAAGAAGAGGCTCGTGTCC-3′ and 5′- GAAAAGTCACCACTTAAGGGGG-3′, described by Yu Y *et al.* [19], and 5′-AGAAGCCTGCACCATGTTTT-3′ and 5′-TGTGGCTTTGCTTTGTCTTG-3′, described by Jira M *et al.* [27], for the *TRPC6* and *AGTR1* genes, respectively.

PCR products were separated by electrophoresis through 3% agarose with ethidium bromide staining. The electrophoresis product was purified using the Nucleic Acid and Protein Purification (NucleoSpin Extract II) kit by Macherey-Nagel (Germany). PCR products were sequenced with the BigDye Terminator version 3.1 Cycle Sequencing Kit by Applied Biosystems (USA) and the reactions were performed on GeneAmp PCR System 2700 by Applied Biosystems (USA). The sequencing reactions were precipitated and finally analyzed on an ABI PRISM 3100 genetic analyzer (Applied Biosystems, USA).

Sequence data was aligned to reference Ensembl DNA sequence [ENSG00000137672], for *TRPC6* gene, and [ENSG00000144891], for *AGTR1* gene, and examined for sequence variations. Detected polymorphisms were confirmed by a second independent PCR reaction and were identified in both forward and reverse strands.

### Statistical analysis

Values are expressed as mean ± SD (standard deviation). Non-parametric test were used to determine if there were significant differences between groups (patients with PAH and controls). Chi-square test was used to compare clinical and hemodynamic variables among genotypes (variables were categorized according to the best cut off point by ROC curve). These correlations were analyzed by the Spearman test. Probability values less than 0.05 were considered statistically significant. Statistical analyses were performed using SPSS for Windows (v19.0).

## Results

### Description of the cohort

**Figure 1:**
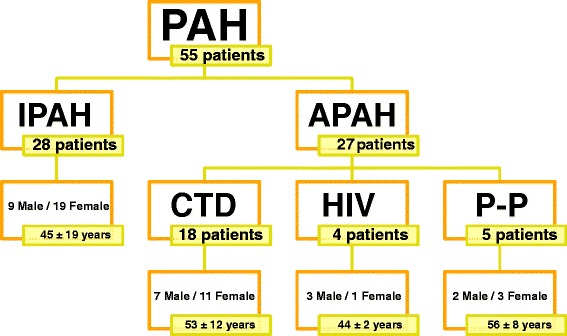
**Disposition of the study population.** This figure shows the total number of patients included in this study separated by PAH type. PAH: Pulmonary Arterial Hypertension; IPAH: Idiopathic Pulmonary Arterial Hypertension; Associated Pulmonary Arterial Hypertension; CTD: connective tissue disease; HIV: Human Immunodeficiency virus; P-P: Porto-pulmonary hypertension.

Fifty five unrelated PAH patients (28 idiopathic, 18 associated to connective tissue disease, 4 related to HIV and 5 porto-pulmonary hypertension) and 52 healthy controls without familial history of PAH were included (Figure 1). At the time of diagnosis 7 patients were in functional class (FC) I, 19 patients in FC II, 25 patients in FC III and 4 in FC IV. Clinical features of patients are shown in Table 1.

**Table 1:** **Clinical features and hemodynamic parameters of patients**

**Clinical features and hemodynamic parameters**	**Clinical data**
***Number***	55
***Age at diagnosis (years)***	49 ± 16
***Gender***	34 F/ 21 M
***mPaP (mmHg)***	48 ± 14
***sPaP (mmHg)***	69 ± 19
***Pulmonary vascular resistance (mmHg.l*** ^***−1***^ ***.m*** ^***−1***^ ***)***	8.3 ± 3.3
***Cardiac index (l.m*** ^***−1***^ ***.m*** ^***−2***^ ***)***	2.9 ± 0.7
***6MWT (m)***	415 ± 146

Values are expressed as mean ± standard deviation; F: female, M: male; mPaP: mean pulmonary pressure; sPaP: systolic pulmonary pressure; 6MWT: 6 minute walking test.

### Analysis of SNPs in the 5′UTR region of TRPC6 gene

**Figure 2:**
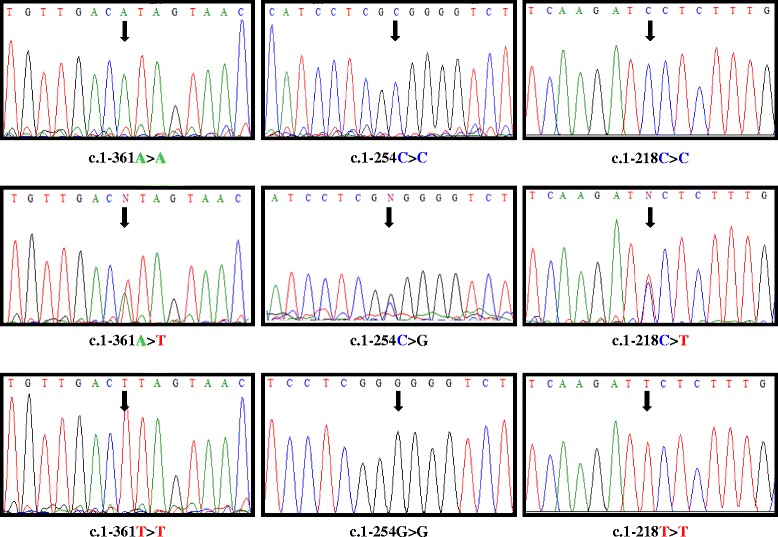
**Representative sequence electropherograms for the 3 variations for** ***TRPC6*** **gene in PAH patients.**

**Figure 3:**
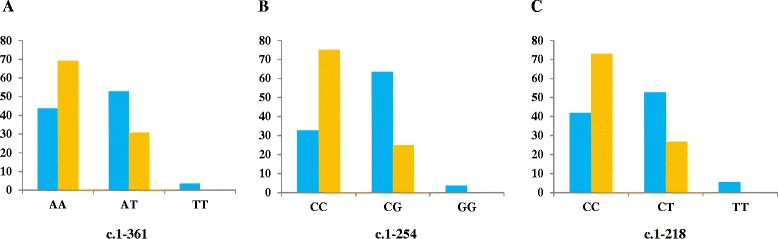
**Genetic disposition between PAH patients (blue) and controls (orange) for each** ***TRPC6*** **SNPs. A**: c.1-361; **B**: c.1-254; **C**: c.1-218.

**Figure 4:**
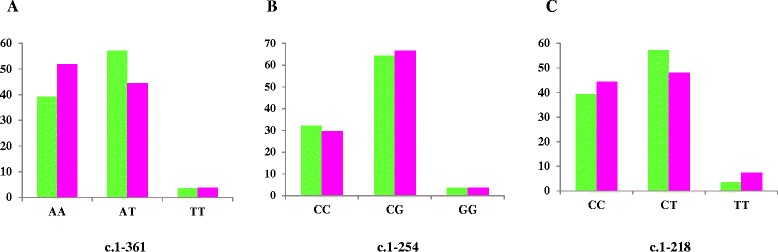
**Genetic disposition of the IPAH (green) and APAH (pink) population for** ***TRPC6*** **gene. A**: c.1-361; **B**: c.1-254; **C**: c.1-218.

We found statistically significant differences between patients and controls (*p* = 0.0077) for *TRPC6* c.1-361A > T polymorphism (Figure 2). TT genotype was present in 2 patients (3.7%) but in any control; AT genotype was also more frequent in patients (52%) than controls (31%), whose most common genotype was AA. These results are shown in Figure 3a. Patients showed an allele frequency of 0.690 for A and 0.303 for T, but this frequency was 0.836 for A and 0.163 for T in controls. Patients with this polymorphism were not in Hardy-Weinberg Equilibrium (H-WE) with p = 0.0085 but controls were in H-WE with p = 0.1588. The predominant genotype for IPAH patients was AT (61%) and for APAH patients was AA (50%) (Figure 4a). Comparing the distribution of this polymorphism, in healthy people reached statistically differences with IPAH (*p* = 0.02), but not with APAH (*p* = 0.097).

The most common genotype in patients for c.1-254C > G polymorphism (Figure 2) was CG (63.7%), while in controls was CC (75%). GG genotype was seen in only 2 patients but not in controls. We found high statistically significant differences between patients and controls for this polymorphism (*p* < 0.0001). Besides, this SNP presented statistical differences between IPAH and controls (p = 0.002) and APAH and controls (p < 0.0001). These results are shown in Figure 3b. Patients were not in H-WE in contrast to controls (p = 0.0037 and p = 0.3029, respectively). The allele frequency for C was 0.645 in patients and 0.875 in controls and for G was 0.354 in patients and 0.125 in controls. For IPAH and APAH patients the most common genotype was GC (64.2% and 60%, respectively) (Figure 4b).

Results for c.1-218C > T polymorphism (Figure 2) are summarized in Figure 3c. For patients, the most common genotype was CT (52.8%), while in controls was CC (73%). TT genotype was seen in 3 patients (5.5%) and any controls. The distribution of this polymorphism reached significant differences between patients and controls (*p* = 0.0021) and between IPAH (most common CT, 57.2%) and APAH (most common CC, 44.5%) (Figure 4c). Both IPAH and APAH patients showed statistical differences with controls (*p* = 0.008 and *p* = 0.014, respectively). Allele frequency for A was 0.609 in patients and 0.836 in controls and for C, 0.390 and 0.163 in patients and controls respectively. Besides, patients (p = 0.0538) and controls (p = 0.6034) were in H-WE.

In this study, 20 of the 55 patients were carriers of the 3 polymorphisms in *TRPC6* gene (36.4%), 11 IPAH (39.2%) and 9 APAH (30%).

### Analysis of the SNP in AGTR1 gene 3′UTR region

**Figure 5:**
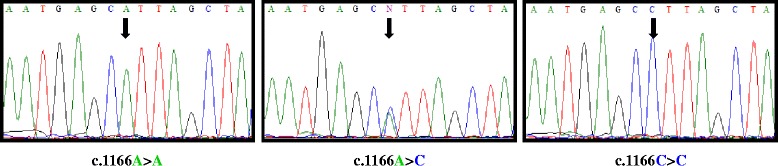
**Representative sequence electropherograms for the 3 variations for** ***AGTR1*** **gene in PAH patients.**

**Figure 6:**
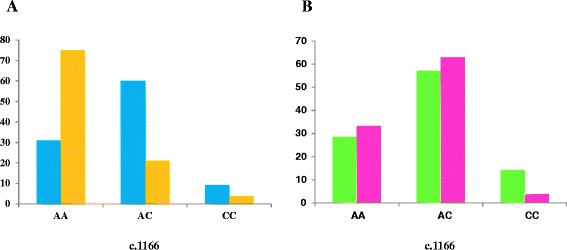
**Genetic disposition of the study population in** ***AGTR1*** **SNP. A**: Show the differences between patients (blue) and controls (orange). **B**: Show the differences between IPAH patients (green) and APAH patients (pink).

We obtained statistically significant differences for c.1166A > C polymorphism (Figure 5), located on 3′UTR of *AGTR1* gene, between patients and controls (p > 0.0001). This SNP was seen in patients with a frequency of 60% in heterozygosis (AC) and 9% in homozygosis (Figure 6a). AC/CC genotype was found in 72.4% of IPAH, and 68% of APAH but only in 25% of healthy people. These two groups (IPAH vs APAH) showed almost no difference between genotypes for this polymorphism (Figure 6b). Both IPAH (*p* > 0.0001) and APAH patients (*p* = 0.001) showed statistical differences with controls. Allele frequency for A was 0.609 in patients and 0.807 in controls, however was 0.391 for C in patients and 0.193 in controls. For this change patients were not in H-WE with p = 0.0063 in contrast to controls with p = 0.1445.

### Correlation with clinical features and hemodynamic parameters

We compared clinical features and hemodynamic parameters between patients according to these SNPs. The variables included were: gender, age at diagnosis, mean pulmonary artery pressure (mPaP), systolic pulmonary artery pressure (sPaP), pulmonary vascular resistance (PVR), cardiac index (CI), 6 minute walking test (6MWT) and type of PAH (IPAH vs APAH). Variables were categorized according to the best cut off point by ROC curve.

Regarding to genotype, several clinical and hemodynamics parameters showed statistically significant differences. For *TRPC6* c.1-361A > T polymorphism and gender (p = 0.002), AT genotype was more frequent in males (78%) and AA genotype in females (56.2%), and patients with AT genotype presented lower CI (p = 0.005). For c.1-254C > G polymorphism we only found statistically differences for CI (p = 0.016), with lower values in carriers of this SNP. For c.1-218C > T polymorphism we also found statistically significant differences in gender (p = 0.016), with CT as predominant genotype in males (65%) and CC females (47%). There were also differences in sPaP (p = 0.039), PVR (p = 0.027) and CI (p < 0.001). Patients carrying this polymorphism had higher values of sPaP and PVR and lower CI (Table 2).

**Table 2:** **Clinical features and hemodynamic parameters of patients with each TPRC6 polymorphism**

**Clinical features and hemodynamic parameters**	**Carriers of c.1-361A > T mutation**		**Carriers of c.1-254C > G mutation**		**Carriers of c.1-218C > T mutation**	
	**Clinical data**	**p-value**	**Clinical data**	**p-value**	**Clinical data**	**p-value**
***Number***	32		37		32	
***Gender***	25 F/7 M	0.002	23 F/14 M	0.177	11 F/21 M	0.035
***Age at diagnosis (years)***	50 ± 16	0.392	48 ± 16	0.403	52 ± 17	0.673
***mPaP (mmHg)***	50 ± 14	0.161	48 ± 14	0.162	48 ± 15	0.307
***sPaP (mmHg)***	71 ± 19	0.190	68 ± 18	0.195	70 ± 20	0.039
***PVR (mmHg.l*** ^***−1***^ ***.m*** ^***−1***^ ***)***	7.8 ± 2.3	0.336	7.6 ± 2.6	0.292	8 ± 2.8	0.027
***CI (l.m*** ^***−1***^ ***.m*** ^***−2***^ ***)***	2.6 ± 0.7	0.005	2.6 ± 0.6	0.016	2.6 ± 0.7	0.000
***6MWT (m)***	400 ± 160	0.136	423 ± 152	0.332	395 ± 141	0.168

For c.1166A > C polymorphism in *AGTR1* gene, carriers showed significant higher values for mPaP (p = 0.035) and PVR (p = 0.035) and lower for CI (p = 0.034) (Table 3). Clinical and hemodynamic parameters did not show any significant differences between associated and idiopathic PAH.

**Table 3:** **Clinical features and hemodynamic parameters in patients with c.1166A > C change**

**Clinical features and hemodynamic parameters**	**Carriers of c.1166A > C mutation**	
	**Clinical data**	**p-value**
***Number***	38	
***Gender***	F/7 M	0.511
***Age at diagnosis (years)***	49 ± 16	0.265
***mPaP (mmHg)***	46 ± 13	0.035
***sPaP (mmHg)***	69 ± 18	0.207
***PVR (mmHg.l*** ^***−1***^ ***.m*** ^***−1***^ ***)***	7.9 ± 2.8	0.035
***CI (l.m*** ^***−1***^ ***.m*** ^***−2***^ ***)***	2.4 ± 0.7	0.034
***6MWT (m)***	413 ± 145	0.138

Patients carrying all 3 polymorphisms showed statistically significant differences in gender (p = 0.016), mPaP (0.011), sPaP (p = 0.040), CI (p < 0.001) and 6MWT (0.049). The combination of these polymorphisms was more frequent in males (59%) than females (41%), with higher values of mPaP, sPaP and PVR, and lower values of CI and 6MWT (Table 4).

**Table 4:** **Clinical features and hemodynamic parameters in patients harboring the 3 SNPs of** ***TRPC6*** **gene and patients with these SNPs plus** ***AGTR1*** **SNP**

**Clinical features and hemodynamic parameters**	**Carriers of c.1-361A > T, c.1-254C > G and c.1-218C > T mutations**		**Carriers of c.1-361A > T, c.1-254C > G, c.1-218C > T and c.1166A > C mutations**	
	**Clinical data**	**p-value**	**Clinical data**	**p-value**
***Number***	19		16	
***Gender***	7 F/12 M	0.016	7 F/9 M	0.135
***Age at diagnosis (years)***	51 ± 17	0.348	53 ± 17	0.049
***mPaP (mmHg)***	51 ± 14	0.011	52 ± 16	0.033
***sPaP (mmHg)***	69 ± 20	0.040	68 ± 22	0.222
***PVR (mmHg.l*** ^***−1***^ ***.m*** ^***−1***^ ***)***	7.4 ± 2.6	0.576	7.2 ± 2.9	0.533
***CI (l.m*** ^***−1***^ ***.m*** ^***−2***^ ***)***	2.5 ± 0.7	0.000	2.7 ± 0.8	0.002
***6MWT (m)***	363 ± 159	0.049	343 ± 165	0.039

The combination of all SNPs in *TRPC6* gene and *AGTR1* gene was detected in 27% of patients analyzed in this study. These patients had a significantly earlier age at diagnosis compared to patients without any SNPs (p = 0.049). Furthermore, these patients showed a higher value of mPaP (p = 0.033) and a lower value for CI (p = 0.002) and 6MWT (p = 0.039). These results are summarized in Table 4.

The mean follow-up period was 14 months. Three patients died during this time (2 APAH, 1 IPAH), therefore it was not possible to analyze mortality. The patient with idiopathic PAH presented the 3 SNPs in *TRPC6* gene and the SNP in *AGTR1* gene. For the two APAH patients, one was a carrier of all 3 SNPs in *TRPC6* gene, and the other patient had c.1-361A > T polymorphism in *TRPC6* gene and the SNP in *AGTR1* gene.

## Discussion

The aim of this study was to analyze three polymorphisms in the 5′UTR region of *TRPC6* gene and another polymorphism in the 3′UTR region of *AGTR1* gene and correlate them with clinical features trying to finding out if they modulate the phenotype of PAH patients. We found statistically significant differences between patients and controls in hemodynamic parameters for these SNPs.

TRPCs play an important role in the cardiovascular system [28]. *TRPC6* is known to be involved in forming receptor-operated Ca^2+^ channels andTRPC6 protein is expressed in lung, brain and smooth muscle cells [29]. In PASMC this protein takes part of the functional Ca^2+^ channels that are activated by vasoconstrictor and mitogenic factors. Enhanced Ca^2+^ influx and elevated [Ca^2+^]_cyt_ are requisites for normal PASMC growth, whereas an excessive Ca^2+^ intake and the subsequent sustained increase in [Ca^2+^]_cyt_ may be critical stimuli for IPAH-PASMC overgrowth [30]. Even in cardiac myocytes, overexpressed *TRPC6* plays an important role in forming Ca^2+^ dependent calcineurin [28]. Downregulation of *TRPC6* inhibits PASMC proliferation [31].

Yu Y *et al*. [19] studied the promoter region of this gene and found that only *TRPC6* c.1-254C > G polymorphism was related to IPAH patients. However, in our study, we found that *TRPC6* c.1-361A > T, c.1-254C > G and c.1-218C > T polymorphisms had different genotype frequency in patients and healthy people, showing significant differences between groups. This could be due to the small sample size more than ethnical differences, since our patient’s cohort has a European background and the allele frequencies described in the control groups are quite similar in the two studies. The study of Yu Y *et al.* showed that allele G in 1-254C > G polymorphism could be responsible for the abnormal *TRPC6* expression and function. The C > G conversion in PAH may alter the regulation of cell cycle progression and proliferation increasing the [Ca^2+^]_cyt_. This channel influx through TRPCs is known to contribute to proliferative mechanisms in PASMC. This SNP showed statistically differences for CI between our patients and controls. Patients carrying the G allele have a lower value, indicating a more severe disease than non-carriers. Perhaps, as Yu Y *et al.* suggest, *TRPC6* c.1-254C > G SNP may predispose individuals to an increased risk of IPAH by linking abnormal *TRPC6* transcription to nuclear factor-κB (NFκB). This fact could be more important for patients with APAH which bear a significant inflammatory burden, as those related to connective tissue diseases, HIV and schistosomiasis, [32]. However, we did not find any differences between IPAH and APAH in our patients.

Regarding to genotype-phenotype correlation, c.1-361A > T polymorphism showed differences in gender, being T allele more frequent in males. We also found a significantly lower CI value in these patients. This data may indicate that this change could have some role in the development of this disease. Similar to c.1-361A > T, c.1-218C > T polymorphism showed significant differences in gender, being more frequent in males, with low CI and higher sPaP and PVR. Carriers of this polymorphism seem to have a more severe disease.

It has been reported that PASMC from patients with IPAH are hyperproliferative, with markedly increased expression of *TRPC6* [28]. PASMC proliferation is regulated by intracellular Ca^2+^. Elevated [Ca^2+^]_cyt_ may induce PASMC proliferation by initiating and promoting the cell cycle, and by triggering or promoting gene transcription via phosphorylation of transcription factors.

For the 254 allele a new binding site for NFκB was created, promoting not also NFκB–mediated *TRPC6* transcription, but perhaps enhancing basal transcription of *TRPC6* in PASMCs [29]. The c.1-254C > G functional study revealed that this SNP enhances NFκB mediated promoter activity and stimulate *TRPC6* expression in PASMCs. Inhibition of NFκB activity attenuated *TRPC6* expression and decreased agonist activated Ca^2+^ influx in PASMCs of IPAH patients harboring the c.1-254G allele [32].Others SNPs in this region could facilitate interactions of several transcription factors (nuclear factor of activated T-cells (NFAT) and Activation Protein-1 (AP-1)) increasing *TRPC6* transcription. Related to it, patients with PAH carrying these polymorphisms could have a worse prognosis than non-carriers. The enhanced transcriptional regulation of *TRPC6* and augmented function of *TRPC6* channels resulting from these polymorphisms may predispose to an increased risk of developing PAH.

Patients with all 3 SNPs in *TRPC6* gene showed statistically significant differences in gender, mPaP, sPaP, CI and 6MWT. Being a carrier of the three polymorphisms was more common in men than in women, even after each SNP was evaluated separately. These patients have higher values of mPaP, sPaP and PVR, and lower values of CI and 6MWT. It seems that carriers of the 3 polymorphisms of *TRPC6* gene present a more severe disease. Although we did not find statistically significant differences in age of diagnosis in our patients, it would be of great interest to know if these 3 SNPs together are related to an earlier onset of the disease.

PAH is more common in women than in men. However, *TRPC6* gene polymorphisms appear more frequently in men. Patients with one or more of these polymorphisms probably are more likely to develop the disease, meaning that this gene could play a more specific role in males. Boney-Montoya *et al*. [33] demonstrated that *TRPC6* expression is not regulated by estradiol in human breast adenocarcinoma cell line (MCF-7) cells, but the pathway of *TRPC6* is still not well understood and perhaps others factors could influence the expression of this channel.

c.1166A > C polymorphism in *AGTR1* gene is a variant extensively investigated in systemic hypertension, but to date only one article has shown a possible role in pulmonary hypertension [25], finding a relation between *AGTR1* polymorphism and age at diagnosis, something that could not be demonstrated in our study. We observed significant differences for this polymorphism between patients and controls. There is enough evidence demonstrating that the binding of angiotensin II to *AGTR1* exerts a pivotal effect on the regulation of vascular tone and salt-water homeostasis. In our cohort the genotype CC + CA was present in 69% of patients and only 25% of healthy. The presence of C allele has been associated with hypertension, aortic stiffness, left ventricular mass and greater coronary artery vasoconstriction [34]. The CC + CA genotypes were associated with a more advanced disease and a significantly higher mortality compared with AA genotype. For *AGTR1* gene,, its potential role in the development of pulmonary hypertension is not well understood, although its function is known from almost 30 years. Li Y *et al*. found that the relation of c.1166A > C polymorphism with coronary heart disease was stronger in older than younger subjects [23]. We detected differences between patients with this SNP for mPaP, PVR and CI. In our series, patients harboring this SNP had a more severe phenotype.

Functional studies have shown that c.1166A > C polymorphism is located in a cis-regulatory site recognized by specific microRNA (miRNA) miR-155 [35,36] which is base-pairing complementary with the 1166A allele but not with the mutant 1166C allele. Jirá M *et al.* described a decrease ability of miR-155 to interact with the cis-regulatory site when this allele is present [27]. Thus, the possibility of failure in the receptor as the biological base is quite likely.

It has been shown that upregulated expression of *TRPM7* (transient receptor potential cation channel, subfamily M, member 7), *TRPV4* (transient receptor potential cation channel, subfamily V, member 4) and *TRPC6* channels in PASMC from IPAH patients seems to be, at least in part, the cause for the enhanced increase in [Ca^2+^]_cyt_ induced by mechanical stimuli (e.g., flow shear stress and stretch). The increase of [Ca^2+^]_cyt_ in PASMC from IPAH patients would contribute to increased pulmonary vascular myogenic tone, sustained pulmonary vasoconstriction and pulmonary vascular medial hypertrophy. Pharmacological blockade of *TRPM7*, *TRPV4* and *TRPC6* channels and/or silent RNA (siRNA) driven downregulation of this channel expression may be a novel therapeutic approach for IPAH patients [37]. In the near future genetic information as this could guide treatment of PAH in a more personalized way.

Obviously, the main limitation for drawing conclusions in our study is the small number of patients, although the low incidence of PAH and some cases that did not consent to be included, did not allow us to have a larger series. The comprehensive study carried out, complete follow-up of all cases and the strength of some findings add value to our results.

## Conclusions

PAH is a rare disease with pulmonary vascular remodeling caused in part by a heterogeneous constellation of genetic arrangements, which ultimately give rise to a common pathologic event, PASMC and endothelial overgrowth. Therefore, the causal and pathogenic mechanism of PAH may involve abnormalities in multiple genes and gene products.

This study, as well as some others previously repported, seems to suggest that c.1-361A > T, c.1-254C > G and c.1-218C > T polymorphisms in *TRPC6* gene and c.1166A > C polymorphism in *AGTR1* could have an important role in the development of this disease and, perhaps, a more severe clinical course.

## Abbreviations

6MWT: 6 minute walking test
*AGTR1*: Angiotensin II type I receptor gene
AP-1: Activation Protein
APAH: Associated pulmonary arterial hypertension
*BMPR2*: Bone morphogenetics protein receptor type 2 gene
CI: Cardiac index
CTD: Connective tissue disease
DNA: Deoxyribonucleic acid
ERS-ESC: European Respiratory Society-European Society of Cardiology
FC: Funcional class
HIV: Human Immunodeficiency virus
HPAH: Inherited pulmonary arterial hypertension
H-WE: Hardy-Weinberg Equilibrium
IPAH: Idiopathic pulmonary arterial hypertension
MCF-7: Human breast adenocarcinoma cell line
miRNA: microRNA
mPaP: Mean pulmonary arterial pressure
NFAT: Nuclear factor of activated T-cells
NFκB: Nuclear factor-κB
PAH: Pulmonary arterial hypertension
PASMC: pulmonary arteriolar smooth muscle cells
PCR: Polymerase chain reaction
PCWP: Pulmonary capillary wedge pressure
P-P: Porto-Pulmonary hypertension
PVR: Pulmonary vascular resistence
SD: Standard deviation
siRNA: Silent RNA
SNP: Single nucleotide polymorphism
sPaP: Systolic pulmonary pressure
*TRPC6*: Canonical transient receptor potential channel isoform 6 gene
*TRPM7*: Transient receptor potential cation channel, subfamily M, member 7 gene
*TRPV4*: Transient receptor potential cation channel, subfamily V, member 4 gene
UTR: Untranslated region
